# Intensive Care Outcomes in Older Adults with and without Dementia: A Systematic Review and Meta-Analysis

**DOI:** 10.1093/geroni/igaf122.3970

**Published:** 2025-12-31

**Authors:** Camila Badell, Maria Jose Lizarazo, Dustin M Solorzano-Salazar, Luis A Figueroa, Katherine K Fung, Eric T Y Chyn, Eloy F Ruiz, Oscar J Ponce-Ponte

**Affiliations:** Rutgers New Jersey Medical School, Lima, Lima, Peru; Mayo Clinic, Minnesota, United States, 3. Knowledge and Evaluation Research Unit, Mayo Clinic, Rochester, Minnesota, United States; Universidad Peruana de Ciencias Aplicadas (UPC), Lima, Lima, Peru; Universidad San Francisco de Quito, Quito, Pichincha, Ecuador; Rutgers New Jersey Medical School, Newark, New Jersey, United States; Rutgers New Jersey Medical School, Newark, New Jersey, United States; Rutgers New Jersey Medical School, Newark, New Jersey, United States; Derriford Hospital, University Hospital Plymouth NHS Trust, Plymouth, England, United Kingdom

## Abstract

Older adults with dementia account over 15% of intensive care unit (ICU) admissions and face higher risks of functional decline and complications. We systematically evaluated the difference in ICU-related outcomes in patients with and without dementia, (e.g., mortality, resuscitation and code status decisions, complications, duration of life support and hospital stay). Searches were performed in MEDLINE, EMBASE, SCOPUS, and Web of Science between 2003 and 2024. A random-effects meta-analysis was done of odds ratios (OR) and mean differences (MD) and their corresponding 95% confidence intervals (CI) were calculated. Fifteen studies, including 1,231,037 patients (12% with dementia), met the inclusion criteria. Adjusted analysis showed that patients with dementia had a higher risk of ICU mortality (OR = 1.05, 95% CI 1.00–1.11), 30-day mortality (OR = 1.54, 95% CI 1.47–1.62) and 12-month mortality (OR = 1.95, 95% CI 1.88–2.02). They were less likely to receive mechanical ventilation (OR = 0.73, 95% CI 0.62–0.85, I2 0.0%) and more likely to develop delirium (OR = 4.80, 95% CI 1.81–12.72). In patients with dementia, ICU stays were slightly shorter (MD = –1 day, 95% CI –1.7 to –0.4), and discharge home was less common (OR = 0.40, 95% CI 0.39–0.42). Older adults with dementia experienced greater risk of mortality and delirium, alongside less aggressive intervention use and healthcare utilization (i.e., shorter ICU stays). These findings underscore the need for prospective studies to optimize ICU decision-making and post-discharge care for this vulnerable population.

